# Supplementary material for: Complexome profiling of the Chlamydomonas psb28 mutant reveals TEF5 as an early PSII assembly factor
Source: Plant Cell. 2025 Mar 17;37(6):koaf055. doi: 10.1093/plcell/koaf055 (PMC12164590; doi:10.1093/plcell/koaf055)
Supplement: koaf055_Supplementary_Data [file koaf055_supplementary_data.zip › psb28_interactive.zip!/plots/intensity/qualityPlots/peptides/HID1_Cre14.g630859.t1.1.html]

### Cre14.g630859.t1.1

Trivial name: HID1  
SeqCoverage [median]: 25.10  
Mass: 40.77 kDa  
  
 

| Sequence | MissedCleavage | Mass [Da] | wtIntensity | muIntensity | Score |
| --- | --- | --- | --- | --- | --- |
| AAAGGGGAGHEAAVPAPPR | 0 | 1612.81 | 588.62 | 2017.55 | 67.42 |
| HLLASGR | 0 | 752.43 | 0.00 | 580.31 | 66.99 |
| IGFVGLGAMGLR | 0 | 1189.66 | 800.24 | 0.00 | 80.44 |
| ILSEAAAAQAAGQGQGQGAPAGEAAAAAGAR | 0 | 2691.33 | 576.07 | 1073.22 | 66.51 |
| QVYLGQAGLLAPK | 0 | 1356.78 | 1443.01 | 7436.01 | 123.63 |
| RLGVDPELMSR | 1 | 1271.67 | 642.01 | 0.00 | 74.61 |
| VLNASSAR | 0 | 816.45 | 2563.74 | 2594.60 | 108.46 |
|  |  |  | 6613.7 | 13701.7 |  |
